# CONTENT ANALYSIS - NURSING HOME (NH) STATEMENTS OF DEFICIENCIES (SODS) AS QUALITY IMPROVEMENT INNOVATION

**DOI:** 10.1093/geroni/igae098.4309

**Published:** 2024-12-31

**Authors:** Mary Dellefield, Caroline Madrigal

**Affiliations:** UCSF, San Francisco, California, United States; VA Boston Healthcare System, Brockton, Massachusetts, United States

## Abstract

The Centers for Medicare and Medicaid (CMS) issue Statements of Deficiency (SODs) collected by trained observers detailing practices that pose harm or ‘immediate jeopardy’ to nursing home residents. SODs document nursing home compliance failures, such as F-tag 0678, which addresses non-compliance with federal cardiopulmonary resuscitation (CPR) requirements, a critical indicator of resident autonomy and self-determination. This study aimed to describe practice patterns and organizational characteristics linked to F-tag 0678 immediate jeopardy citations in 80 nursing homes across CMS Region 5 (Michigan [17], Minnesota [30], Ohio [22], Wisconsin [11]} from 2019-2022. An a-priori code book was developed, based on previous SOD research and American Heart Association guidelines. Content analysis was applied to SODs to identify practice patterns and organizational characteristics. Sampled nursing homes were for-profit (69%), with a mean bed size of 93 (range= 26-212) and an average star rating of 2.4 (range=1-5). Major practice pattern failures included: early detection (identifying code status/initiating CPR); logistics (communication failures; failures to follow policy/procedures); documentation (lack of clarify about code status/inaccurate documentation of CPR event); staff performance (lack of familiarity with nursing home operations); and delay in initiating CPR (13 minutes to 4 hours). Structured analysis of SODs offers a novel, efficient approach to leverage existing CMS data to drive quality improvement efforts and inform policy decisions. This research has the potential to inform the development of evidence-based interventions, enhance regulatory oversight, improve the quality of care, and protect the rights of vulnerable nursing home residents.

